# The mitochondrial genome of *Phallusia mammillata *and *Phallusia fumigata *(Tunicata, Ascidiacea): high genome plasticity at intra-genus level

**DOI:** 10.1186/1471-2148-7-155

**Published:** 2007-08-31

**Authors:** Fabio Iannelli, Francesca Griggio, Graziano Pesole, Carmela Gissi

**Affiliations:** 1 Dipartimento di Scienze Biomolecolari e Biotecnologie, Università di Milano, Via Celoria 26, 20133 Milano, Italy; 2 Dipartimento di Biochimica e Biologia Molecolare "E. Quagliariello", Università di Bari, Via Orabona 4, 70126 Bari, Italy

## Abstract

**Background:**

Within Chordata, the subphyla Vertebrata and Cephalochordata (lancelets) are characterized by a remarkable stability of the mitochondrial (mt) genome, with constancy of gene content and almost invariant gene order, whereas the limited mitochondrial data on the subphylum Tunicata suggest frequent and extensive gene rearrangements, observed also within ascidians of the same genus.

**Results:**

To confirm this evolutionary trend and to better understand the evolutionary dynamics of the mitochondrial genome in Tunicata Ascidiacea, we have sequenced and characterized the complete mt genome of two congeneric ascidian species, *Phallusia mammillata *and *Phallusia fumigata *(Phlebobranchiata, Ascidiidae). The two mtDNAs are surprisingly rearranged, both with respect to one another and relative to those of other tunicates and chordates, with gene rearrangements affecting both protein-coding and tRNA genes. The new data highlight the extraordinary variability of ascidian mt genome in base composition, tRNA secondary structure, tRNA gene content, and non-coding regions (number, size, sequence and location). Indeed, both *Phallusia *genomes lack the *trnD *gene, show loss/acquisition of DHU-arm in two tRNAs, and have a G+C content two-fold higher than other ascidians. Moreover, the mt genome of *P. fumigata *presents two identical copies of *trnI*, an extra tRNA gene with uncertain amino acid specificity, and four almost identical sequence regions. In addition, a truncated cytochrome b, lacking a C-terminal tail that commonly protrudes into the mt matrix, has been identified as a new mt feature probably shared by all tunicates.

**Conclusion:**

The frequent occurrence of major gene order rearrangements in ascidians both at high taxonomic level and within the same genus makes this taxon an excellent model to study the mechanisms of gene rearrangement, and renders the mt genome an invaluable phylogenetic marker to investigate molecular biodiversity and speciation events in this largely unexplored group of basal chordates.

## Background

The mitochondrial genome (mtDNA) is considered a model system for studying genome evolution, and a good molecular marker to reconstruct the phylogeny of Metazoa, both at high and low taxonomic level. In general, evolutionary genomics and phylogenetic studies are strictly related, because the understanding of the evolutionary peculiarities of a given character greatly facilitates the evaluation of its suitability as phylogenetic marker. For phylogenetic purposes, the mitochondrial sequences of single or multiple genes or regions are commonly analysed by traditional molecular evolutionary methods. However, besides the sequence itself, genome-level features have been proposed as powerful phylogenetic characters [1]. Gene arrangement, gene content, tRNA secondary structures, and changes in genetic code are all mitochondrial genome-level characters, used to clarify some controversial phylogenetic relationships, especially at high taxonomic levels. However, the phylogenetic reliability of these characters needs to be investigated more in detail, considering the variability of the mitochondrial genome in metazoans.

The mtDNA of Metazoa is a small circular genome, of around 14–16 kb in length, characterized by a compact structure with no introns and short intergenic regions. Only one large non-coding region is typically present in the genome and is known as the control region (CR), because it is assumed that elements controlling replication and transcription of the genome are located in this region, although functional studies have been carried out only in vertebrates and a few other species [2-4]. In most invertebrate mtDNAs where only short non-coding regions are present, the CR has sometimes been tentatively identified through the presence of repeated sequences, secondary structures, or a strong compositional bias, because these features are often associated with the control region [5,6]. The mt gene content is extremely conserved and typically consists of 37 genes encoding two rRNAs, 22 tRNAs, and 13 proteins, all of which are components of the respiratory chain complexes. Variations in gene content are rarely due to changes in the protein-coding gene complement, and mostly concern changes in the content of tRNA genes. Indeed, only the *atp8 *gene, encoding for subunit 8 of ATP synthase, is lost in Platyhelminthes [7], and in most mollusc Bivalvia [8] and Nematoda [9]. On the contrary, drastic changes in tRNA gene number are related to the usage of a modified genetic code [10,11] or are restricted to specific taxonomic groups. Thus, the mtDNA of Cnidaria encodes only for one or two tRNAs (tRNA-Met and/or -Trp) [12,13], whereas the mtDNA of Porifera usually present extra and/or duplicated tRNAs in variable number, but can also have an incomplete tRNA set [14,15]. In addition, the loss or duplication of one or few tRNA genes occurs sporadically in many taxa, sometimes associated with changes in gene order. Mitochondrial gene arrangement varies both across phyla and within a given phylum, and exhibits a high degree of variability in invertebrates [16], while the vertebrate gene order is almost frozen and highly similar to that of lancelets (Cephalochordata) [17]. Extensive gene rearrangements have been observed in Mollusca [5], Nematoda [18], and some arthropod lineages such as hemipteroid insects [19,20] and several parasitic groups [20-23]. Although gene order is generally conserved at low taxonomic levels, such as within a genus or a family, exceptions have been reported in the most diverse taxonomic groups, even those commonly characterized by a stable gene order. For example, within Vertebrata, salamanders of the family Plethodontidae show extensive gene rearrangements, moreover the presence of pseudogenes and additional gene copies testifies to ancient duplications of large mt regions [24]. Similarly, lancelets of the genus *Epigonichthys *shows an inversion and several gene translocations, explained by recombination and tandem duplications, respectively [17]. Genome rearrangements at intra-genus level have been observed in Mollusca and Arthropoda, such as in marine gastropods of the genus *Dendropoma *[25], bivalves of the genus *Crassostrea *[26], and even between gender-specific mtDNAs of bivalves with doubly uniparental inheritance (DUI). In particular, the extent of rearrangements in DUI mitotypes ranges from several gene inversions, duplications and transpositions in *Inversidens japanensis*, to an almost-identical or identical gene order in *Tapes philippinarum *and *Mytilus galloprovincialis *[27,28]. Among arthropods, chigger mites of the genus *Leptotrombidium *(Acari) show translocation of a tRNA gene and duplications of non-coding and rRNA sequences, in addition to an overall gene order drastically different from the arthropod ancestral gene order [29]. Finally, parasitic flatworms of the genus *Schistosoma *exhibit extensive rearrangements due to translocations of protein-coding and tRNA genes [30,31].

The investigation of gene rearrangements in closely related species, such as congeneric species, can be extremely useful to discover the mechanisms underlying gene order variability, as it allows the observation of recent rearrangements and even the identification of intermediary stages of the process itself. To date, the mtDNA of more than 1000 metazoan species has been sequenced but the taxon sampling is highly biased towards vertebrates and arthropods (771 and 133 species, respectively), with few or no complete genome sequences available for many taxonomic groups (GenEmbl, July 2007). The availability of congeneric mt genomes is also extremely limited. Within Tunicata, traditionally considered the basal subphylum of Chordata (but see also [32]), the mtDNA has been completely sequenced only in one representative of the class Thaliacea (*Doliolum nationalis*) and four representatives of the class Ascidiacea, including three species of the genus *Ciona*. Surprisingly, substantial gene rearrangements have been observed within the genus *Ciona *[33], and have significantly contributed to demonstrate the existence of two cryptic species in *Ciona intestinalis *[34]. Moreover, ascidian mt genomes show several unusual features compared to other chordates, such as a modified genetic code [10], a fast nucleotide substitution rate [35,36], a gene order extremely rearranged compared to other chordates and metazoans [33,36], the presence of two distinct tRNA-Met genes [37], and the absence of a discernible control region [33]. These features suggest that further investigations on tunicate mtDNA should be of interest. Indeed, the highly variable gene order observed even at intra-genus level in the currently limited tunicate sample suggests that this taxon may be a good model for the investigation of mechanisms of genome rearrangement. Moreover, the unexpected evolutionary dynamics of the mtDNA of tunicates suggests caution in the usage of this molecule to reconstruct the phylogeny of Chordata, which is in turn a prerequisite to understand the origin of Vertebrata [32]. As first step to verify the reliability of tunicate mtDNA as phylogenetic marker in chordates, it is fundamental to increase the number of available sequences.

To investigate the variability of ascidian mtDNA at low taxonomic levels, we have sequenced the complete mtDNA of the two congeneric ascidians *Phallusia fumigata *and *Phallusia mammillata*, and carried out detailed comparisons of the structural and evolutionary features of ascidian mtDNAs at the intra-genus level. The *Phallusia *genus was chosen for this study as it belongs to the same order as *Ciona *(order Phlebobranchiata) but to a different family (Ascidiidae versus Cionidae), thus the divergence between these organisms is adequate to confirm whether the intra-genus evolutionary dynamics observed in *Ciona *are a common ascidian trait.

## Results and discussion

### Gene content

The mitochondrial genome of both *Phallusia *species contains the canonical set of two rRNA genes (*rrnS *and *rrnL*) and 13 protein-coding genes, including the *atp8 *gene previously considered absent in tunicates [35]. The complement of tRNA genes is unusual, being different between the two congeneric species and with respect to other ascidians. In accordance with the tunicate mt genetic code [10,35], two tRNA genes for Leu, Ser, and Gly codons have been identified. In addition, tunicate mtDNAs specify two tRNA-Met, one with the common 5'-CAU-3' and the other with the unusual 5'-UAU-3' anticodon, thus the canonical tRNA set for tunicates consists of 24 tRNAs [33,37]. Both tRNA-Met genes have been found in *Phallusia *mtDNAs, confirming the hypothesis that this is a general feature of ascidian mtDNA. However, the tRNA-Asp gene (*trnD*) is lost in both genomes, and in *P. fumigata *two extra tRNA genes have been identified: an additional gene for tRNA-Ile (*trnI-2*), and a *trnX *gene that can be folded into two alternative cloverleaf structures showing different anticodons (see paragraph on tRNA genes). The two copies of *trnI *are almost identical, a situation similar to that found in the mtDNA of the distantly related species *Halocynthia roretzi*, where there are two almost identical copies of *trnF *[38]. In conclusion, 23 tRNA genes are present in the mtDNA of *P. mammillata*, and 25 in *P. fumigata*.

### Genome size and compactness

The mtDNAs of *P. mammillata *and *P. fumigata *are 14579 and 15535 bp long respectively, which is consistent with the typical size of chordate mt genomes. Surprisingly, the mtDNA of *P. mammillata *is 6.5% (956 bp) shorter than that of *P. fumigata*, a somewhat unexpected observation give the almost identical size of the mtDNA of the three *Ciona *species (maximum difference: 1.4% – 199 bp – between *C. intestinalis sp. A *and *sp. B*).

The more compact structure of the *P. mammillata *mtDNA compared to *P. fumigata *is highlighted not only by the smaller genome size but also by the higher number/percentage of gene overlaps, and the lower percentage of non-coding (NC) sequences. As reported in Table 1, overlapping gene pairs account for 44 bp in *P. mammillata*, and 23 bp in *P. fumigata *(0.30 against 0.15% of the total genome). Thus, there are six and three overlapping gene pairs in *P. mammillata *and *P. fumigata*, respectively, with only two overlapping gene pairs common to both species (*cox2*-*cob *and *trnL(CUN)*-*trnT*). In parallel, the percentage of NC sequences is quite low in the more compact mtDNA of *P. mammillata *(1.94%) and higher in *P. fumigata *(5.89%) (Table 2). Distinct from the *Phallusia *genus, the three species of *Ciona *reveal almost identical values of gene overlap (Table 1), and a notably lower %NC only in *C. intestinalis sp. B *(Table 2) [34]. Overall, the involvement of both NC total size and gene overlaps in the determination of mtDNA compactness is demonstrated by the highly significant negative correlation between these parameters in a comparative analysis between all the ascidian mtDNAs (r = 0.9).

**Table 1:** Statistics on gene overlaps of ascidian mitochondrial genomes

	**Gene overlap**			**Longest overlap**	
		
**Species^a^**	**%**	**Total bp**	**N° (L ≥ 1)**	**bp**	**Gene pair**
*Phallusia mammillata*	0.30	44	6	14	*cox2-cob*
*Phallusia fumigata*	0.15	23	3	14	*cox2-cob; nad1-nad2*
*Ciona intestinalis sp. A*	0.26	37	3	26	*cox2-cob*
*Ciona intestinalis sp. B*	0.25	38	3	29	*cox2-cob*
*Ciona savignyi*	0.22	33	3	26	*cox2-cob*
*Halocynthia roretzi*	0.23	34	4	13	*nad4L-trnM(CAT)*

^a ^Sequence AC numbers are reported in Additional file 2.

**Table 2:** Statistics on non-coding (NC) sequences of ascidian mitochondrial genomes

	**% NC (bp)**	**N°^b^**	**NC length distribution**			**Longest NC**	
			
**Species^a^**			**20–39 bp**	**40–99 bp**	**> 99 bp**	**bp**	**Location^c^**
*Phallusia mammillata*	1.94 (283)	21	0	3	0	65	*trnC-nad4L*
*Phallusia fumigata*	5.89 (915)	25	2	2	5	134	*cox1-trnG(GGN)*
*Ciona intestinalis sp. A*	2.9 (429)	22	2	4	0	85	*nad1-rrnL*
*Ciona intestinalis sp. B*	1.8 (263)	23	1	2	0	71	*cox3-trnK*
*Ciona savignyi*	2.9 (428)	25	1	1	1	194	*nad1-trnP*
*Halocynthia roretzi*	3.09 (456)	29	5	1	1	112	*nad4-trnV*

^a ^Sequence AC numbers are reported in Additional file 2.

^b ^Number of non-coding sequences, including spacers 1 bp long.

^c ^The location of NC sequences is defined by flanking genes.

In conclusion, different evolutionary trends toward compactness have shaped the mtDNAs of the *Phallusia *genus, giving rise to substantial differences in genome size between *P. mammillata *and *P. fumigata*. By way of contrast, the compactness is almost the same in the three mtDNAs of the genus *Ciona*, where mtDNA size variability is mainly due to differences in NC size [34].

### Non-coding regions

No single non-coding region comparable in size to the control region of vertebrates is found in the mtDNA of the two *Phallusia *species.

NC sequences constitute 21–25 short intergenic regions (L < 200 bp), and the NC size distribution is quite different between the two species (Table 2). Indeed, five NC longer than 100 bp are present in *P. fumigata *but none in *P. mammillata*. Moreover, variability in NC size distribution is common to all other analysed ascidians (Table 2). The largest NC region is more than two times longer in *P. fumigata *compared to *P. mammillata*, as observed in *C. savignyi *compared to the two *C. intestinalis *species (Table 2).

The position of NC regions longer than40 bp is not conserved in ascidians. Indeed, only one NC region is located exactly in the same position in the two *Phallusia *species (NC region between *trnC*-*nad4L*). Similarly, only one NC region is located in a similar but not identical position in the three available *Ciona *species (NC region downstream *nad1*).

No significant sequence similarity has been found between NC sequences of the two *Phallusia *species, neither between any of these NC and those of other ascidians.

**Figure 1:**
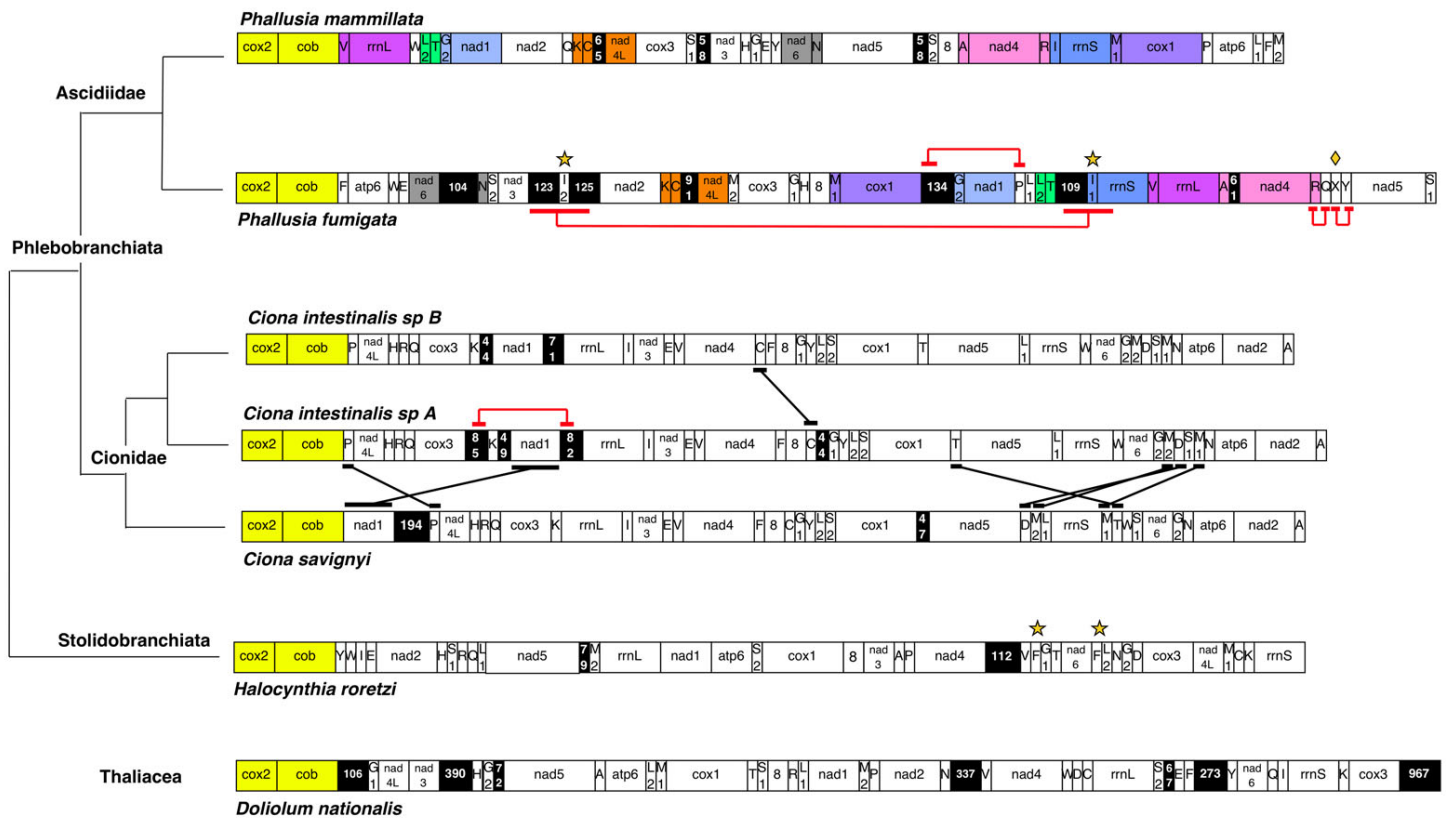
**Gene organization of tunicate mitochondrial genomes, and taxonomic classification of the analysed species**. Gene blocks conserved between *P. mammillata *and *P. fumigata *are reported in different colours, with yellow indicating the gene pair conserved in all analysed tunicates. Sequences almost identical in the same mtDNA are underlined and linked by a red line. Genes transposed among the three *Ciona *species are underlined and linked by a black line. Non-coding (NC) regions equal or longer that 40 bp are indicated by black background, with numbers corresponding to their size (in bp). Abbreviations for protein-coding and rRNA genes are as in the main text, except for *atp8 *gene (abbreviation: 8). Transfer RNA genes are indicated according to the transported amino acid, except for: G1: Gly(AGR); G2: Gly(GGN); L1: Leu(UUR); L2: Leu(CUN); M1: Met(AUG); M2: Met(AUA); S1: Ser(AGY); S2: Ser(UCN). Diamond: *trnX *gene of *P. fumigata*. Star: intra-genome duplicated genes, that is *trnI *genes in *P. fumigata *(named I1 and I2), and *trnF *in *Halocynthia roretzi*. Feature table of *Halocynthia roretzi *mtDNA is as reported in [38].

**Figure 2:**
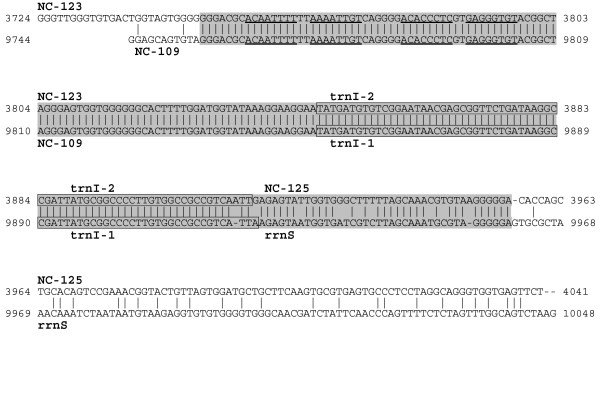
**Similarity between the two *trnI *sequences of *P. fumigata*, including flanking non-coding (NC) regions**. A gray background highlights the 208 bp sequence with 95% identity between the two regions, with *trnI-1 *and *trnI-2 *sequences boxed. Gene abbreviations are as in the main text. Non-coding regions are named according to their length (in bp). Underlined sequences indicate inverted repeats.

**Figure 3:**
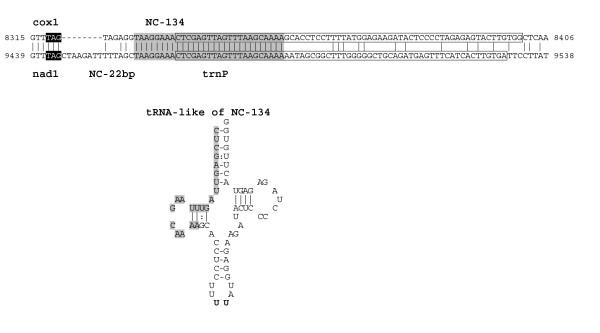
**Similarity between the non-coding region 134 bp-long (NC-134) and the *trnP *gene of *P. fumigata*, together with the tRNA-like structure assumed by NC-134**. A gray background indicates the 29-bp identical sequences, and boxed sequences identify tRNA and tRNA-like structures. In the tRNA-like structure, canonical and G-U base pairing are differently indicated. Stop codons are in black background.

Sequence similarities between NC of the same mtDNA have been found only in *P. fumigata *(see Figure 1, where NC are named according to their size). In particular, the block NC(123 bp)-*trnI2*-NC(125 bp) shows a 95% identity over 208 bp to the block NC(109 bp)-*trnI1*-*rrnS*(first 38 bp), including an identical sequence 165 bp long (Figure 2). The NC(134 bp) located between *cox1 *and *trnG(GGN) *contains a sequence 29 bp-long duplicated in the region downstream *nad1 *(Figure 3). Moreover, this 29 bp-long sequence constitutes a repeat located upstream and downstream of the gene pair *trnG(GGN)-nad1*, similarly to the situation found in *C. intestinalis sp. A*, where a completely different sequence, 25 bp-long, flanks the gene pair *trnK*-*nad1 *[33]. Thus, in both *C. intestinalis sp. A *and *P. fumigata*, a completely different duplicated sequence is located upstream and downstream a gene pair including a tRNA gene (*trnK *or *trnG-GGN*) and *nad1*. These sequences can be explained as remnants of gene rearrangement events involving *nad1 *and occurred independently in the two species, or as sequences that promoted the rearrangement event. Alternatively, they could also be unknown functional sequences involved in the regulation of *nad1 *expression.

Additional elements characterize the long NC regions of *P. fumigata*: two palindromes 8 bp long are found in the NC upstream of the duplicated *trnI *genes (see Figure 2); a tRNA-like secondary structure, with sequence similarity to the beginning of tRNA-Pro, has been found in the NC-134 bp (Figure 3). It is striking that palindromes or tRNA-like structures have often been identified in the control region of metazoans, which suggests they may be involved in the mt replication or transcription processes.

In conclusion, NC regions appear highly variable in number, size, sequence and location in the *Phallusia *as in the *Ciona *genus suggesting that a high variability in NC is quite common between closely related ascidian species. The extensive reorganization of NC regions could partially be related to the extensive changes in gene order, indeed a process of "duplication-random gene loss" can generate NC fossil sequences, as remnants of duplicated genes erased by evolution.

### Gene order

In both *Phallusia *species all genes are transcribed from the same strand, as in other tunicates so far studied.

Gene order is extremely rearranged between *P. mammillata *and *P. fumigata*: only nine gene blocks, including about half of the total mitochondrial genes, are conserved in both species, and the largest of these blocks consists of only three genes (Figure 1). The breakpoint analysis confirms this observation (Table 3), as the relative breakpoint distance is very high and quite close to the value expected for random permutations (0.99). Moreover, gene rearrangements are not due to a high mobility of tRNA genes, indeed omitting the tRNAs the relative breakpoint distance between *Phallusia *species increases (Table 3) and only two gene blocks appear conserved (*nad2*-*nad4L*-*cox3*, and the previously identified *cox2*-*cob *gene pair, accounting for one-third of non-tRNA genes).

**Table 3:** Relative breakpoint distance and sequence divergence for several intra-genus pairs of protochordates

	**Genus**	**Species-1**	**Species-2**	**Breakpoint^a^**		**Protein-genes^b^**		**Protein**	**rRNA^d^**	
							
				**All**	**-tRNA**	**dN**	**dS**	**% id^c^**	***rrnS***	***rrnL***
Ascidiacea	*Phallusia*	*mammillata*	*fumigata*	0.71	0.80	0.182	56.60	72.36	0.452	0.412
	*Ciona*	*intestinalis sp. A*	*intestinalis sp. B*	0.08	0	0.055	3.25	88.79	0.113	0.147
	*Ciona*	*intestinalis sp. A*	*savignyi*	0.33	0.20	0.156	129.21	72.66	0.403	0.295
	*Ciona*	*intestinalis sp. B*	*savignyi*	0.41	0.20	0.169	83.0	71.42	0.396	0.310
Cephalochordata	*Branchiostoma*	*floridae*	*lanceolatum*	0	0	0.087	3.12	85.84	0.269	0.287
	*Branchiostoma*	*belcheri*	*lanceolatum*	0	0	0.097	3.94	84.26	0.257	0.236
	*Branchiostoma*	*floridae*	*belcheri*	0	0	0.110	3.75	82.54	0.289	0.263
	*Epigonichthys*	*lucayanus*	*maldivensis*	0.22	0.33	0.171	10.29	74.87	0.405	0.402

^a ^Relative breakpoint distance calculated dividing the pairwise breakpoint distance by the number of genes shared by corresponding genomes, for a dataset including all mt genes (All), and a dataset without tRNA genes (-tRNA).

^b ^dN (nonsynonymous substitutions) and dS (synonymous substitutions) calculated on the 13 mt protein-coding genes according to the codon substitution model of Goldman and Yang [73].

^c ^Percentage of amino acid identity calculated on the 13 mt-encoded proteins

^d ^Substitutions per sites calculated according to the GTR model [75].

Gene rearrangements in the *Phallusia *appear to be more extensive than those observed in the *Ciona *and in other congeneric protochordate pairs (Table 3). As reported in Figure 1, only the tRNA-Cys gene (*trnC*) is translocated in *C. intestinalis sp. A *compared to *C. intestinalis sp. B *[34], whereas between *C. intestinalis sp. A *and *C. savignyi *there are six translocated genes (*nad1 *and five tRNA genes) [33]. Thus, the ancestral gene order state could be reconstructed quite easily for the *Ciona *genus but not for *Phallusia*, where several duplication/transposition steps will be required to relate the two gene orders. On the contrary, gene order is almost invariant in lancelets (Table 3), with translocations and inversions found only in the genus *Epigonichthys*, specifically only in *E. lucayanus *compared to all other lancelets studied [17].

Gene order comparisons among all tunicate mtDNAs, both with and without tRNA genes, do not identify large gene blocks shared by most species. Thus, including all genes, only *cox2*-*cob *is conserved in all tunicates, probably because of the ORF overlap (see below), and *trnR*-*trnQ *is conserved in almost all ascidians except for *P. mammillata *(Figure 1). Excluding tRNA genes, no additional or largest gene blocks appear conserved in all tunicates or ascidians, and only *nad4L*-*cox3 *is shared by the order Phlebobranchiata (i.e. *Ciona *and *Phallusia *genera).

The absence of a consensus gene order for ascidian mtDNA complicates comparisons with the almost frozen gene order of vertebrates and cephalochordates [16,17]. The few instances of gene blocks shared by tunicates and other-chordates appear due to fortuitous similarities, rather than to a common evolutionary origin, as indicated by their presence in only one or few tunicates. For example, the *trnV*-*rrnL *gene pair is shared by vertebrates, cephalochordates, and *Phallusia *species (Phlebobranchiata), but it is absent in all remaining tunicates including other phlebobranches of the genus *Ciona *(see Figure 1). Similarly, the gene block *rrnS*-*trnV*-*rrnL *is shared only by vertebrates and *P. fumigata*. Although these gene blocks are quite primitive, as indicated by their presence even in other deuterostomes, their occurrence only in the *Phallusia *genus among all tunicates suggests that this may be the result of multiple gene rearrangements, rather than the remnant of a primitive gene order.

Although complete mtDNA sequences are still underrepresented for ascidians, it is evident that gene rearrangements are frequent in this group, and quite common at low taxonomic levels at least in the order Phlebobranchiata. It should be verified if intra-genus rearrangements are frequent even in ascidians belonging to the remaining orders of Stolidobranchiata and Aplousobranchiata. The evolutionary trend of ascidians toward gene rearrangements is completely different from other chordates, and even from other metazoans. In most metazoans, mitochondrial gene order has been demonstrated to evolve in a non-clocklike, saltatory manner, and changes are commonly described as rare, unique events unlikely to be subjected to reversion or convergent evolution [1,16,39]. Therefore, this genome-level character has been considered highly reliable as a phylogenetic marker (review in [1]). In ascidians, the frequent gene rearrangements could impair the reliability of this feature as a phylogenetic marker. This high frequency of rearrangement could also imply differences in the ascidian gene rearrangement mechanisms compared to other metazoans. Gene rearrangements are commonly explained by a process of "tandem duplication-random gene loss" [40], assuming that duplicated portions of the mtDNA can be generated by several processes, and that supernumerary copies of each gene will be selected at random to be subsequently eliminated [41]. A few cases of mt gene rearrangements have been explained by different mechanisms, such as a tandem duplication process followed by non-random gene loss based on gene transcriptional polarities, suggesting that functional constraints could overcome the random process of gene loss [42]. Finally, intra- and inter-genome illegitimate recombination has also been observed [43], or hypothesized [44] in organisms such as nematodes and mites. As simple speculation, the high frequency of rearrangements in ascidians could be due to a completely different mechanism, or to the exacerbation of one of the previously hypothesized mechanisms (i.e. due to an increased activity of enzymes already involved in the process or due to the acquisition of new enzymatic activities). The "duplication-random gene loss" model does not fit well the gene rearrangements of the *Phallusia *genus, because a large number of gene movements are required to explain the observed gene order, however it is still compatible with the situation found in the *Ciona*. Thus, the high frequency of gene rearrangements observed in ascidians could be partially related to the existence of long divergence times between morphologically closely related species. Moreover, the absence of data on the replication process and the location of control region in ascidian mtDNA prevents further speculations on the rearrangement process. Indeed, in most metazoan mtDNAs the replication origins act as hot spots for gene rearrangements [16], suggesting that components of the replication machinery are involved in the step of segmental duplication.

### Sequence divergence

Table 3 reports the sequence divergence between the two *Phallusia *species and other congeneric pairs of protochordates, calculated separately for rRNA genes, synonymous (dS) and nonsynonymous sites (dN) of protein-coding genes, and amino acid sequences. As expected, dN values and percentage of amino acid identity are negatively correlated (r = 0.98), and a strong positive correlation is observed between dN and rRNA sequence divergence (r = 0.97 and 0.88 for *rrnS *and *rrnL*, respectively), indicating that different mt genes follow the same evolutionary trends. Synonymous substitutions appear saturated in all intra-genus comparisons, as indicated by dS values higher than one (Table 3). Both nonsynonymous sites and rRNA genes indicate a sequence divergence between *P. mammillata *– *P. fumigata *highly similar to that observed between lancelets *E. lucayanus *– *E. maldivensis*, and in comparisons between *C. intestinalis *– *C. savignyi*. Assuming a constant evolutionary rate, a similar divergence time can be inferred for the above-mentioned species pairs, whereas a more recent divergence can be hypothesized for the species pair *C. intestinalis sp. A *– *C. intestinalis sp. B*, which shows the lowest dN and rRNA divergence values (Table 3).

In the protochordate sample here analysed, there is no statistically significant correlation between the relative breakpoint distance and the evolutionary rate of rRNA or protein-coding genes, although in arthropods a general correlation between the rate of genome rearrangement and the rate of nucleotide substitution has been found [5,45]. However in our small sample, species pairs with the highest gene rearrangements also exhibit the highest sequence divergence in both Cephalochordata and Ascidiacea (Table 3).

Considering the single mitochondrial-encoded proteins, COX1 is the most conserved protein (93.8% identity in *Phallusia*), whereas ATP8, NAD6 and NAD2 are among the less conserved proteins in all analysed ascidians (NAD6 is the least conserved protein in *Phallusia*, with 45.1% identity).

### Base composition

As reported in Table 4, the G+C content of *P. mammillata *and *P. fumigata *mtDNAs is significantly higher than that of other ascidians (average values 46.9 ± 0.2% in *Phallusia*, and 24.8 ± 4.7 in other ascidians). The trend toward a GC-rich genome is even more pronounced at the third codon position (P3) of protein-coding genes, where the G+C content is 51.5 ± 1.1% in *Phallusia *and 15.8 ± 5.9% in remaining ascidians. This high G+C content is due to the strong increase of G in *Phallusia *compared to remaining species (Table 4). Indeed, in other ascidians the most common base is T, and the T/G ratio is always higher than one, ranging from 2.4 to 8.2 in P3. On the contrary, in both *Phallusia *species the G content is almost as high as the T content, thus the T/G ratio is close to one and becomes lower than one at P3 in *P. fumigata*. At intra-genus level, base composition is constant in *Phallusia *and quite variable in *Ciona*, as shown by the standard deviation of the average GC content calculated on P3 in intra-genus comparisons (51.5 ± 1.1% in the *Phallusia *genus and 13 ± 2.5% in the *Ciona *genus).

**Table 4:** Compositional features of tunicate mitochondrial genomes

	**G+C%**		**T/G ratio**		**%T**		**%G**		**%A**		**%C**		**GC-skew**		**AT-skew**	
	
	**All^a^**	**P3^b^**	**All**	**P3**	**All**	**P3**	**All**	**P3**	**All**	**P3**	**All**	**P3**	**All**	**P3**	**All**	**P3**
**Ascidiacea**																
*Phallusia mammillata*	47.1	50.7	1.18	1.12	33.3	32.1	28.2	28.6	19.5	17.2	18.9	22.1	0.20	0.13	-0.26	-0.30
*Phallusia fumigata*	46.8	52.2	1.07	0.86	33.3	30.7	31.1	35.5	19.9	17.1	15.7	16.7	0.33	0.36	-0.25	-0.29
*Ciona intestinalis sp. A*	21.4	10.5	3.72	8.18	44.4	49.3	11.9	6.0	34.2	40.2	9.5	4.4	0.12	0.15	-0.13	-0.10
*Ciona intestinalis sp. B*	23.5	15.5	3.47	5.95	43.5	46.9	12.5	7.9	33.1	37.6	10.9	7.7	0.07	0.01	-0.14	-0.11
*Ciona savignyi*	22.7	13.0	3.22	4.99	45.3	51.9	14.1	10.4	32.0	35.2	8.7	2.6	0.24	0.61	-0.17	-0.19
*Halocynthia roretzi*	31.7	24.1	1.90	2.38	44.0	50.0	23.2	21.0	24.3	26.0	8.5	3.1	0.46	0.74	-0.29	-0.32
**Thaliacea**																
*Doliolum nationalis*	39.0	39.4	1.64	1.74	32.8	30.9	20.0	17.8	28.3	29.7	19.0	21.7	0.03	-0.10	-0.07	-0.02

^a ^All: whole mitochondrial genome.

^b ^P3: third codon position of the 13 protein-coding genes.

An asymmetric distribution of complementary bases between the two strands is observed in all ascidians: on the coding strand, the GC-skew is positive and the AT-skew is negative, with absolute values of both skews quite low in most species (Table 4). Surprisingly, the compositional asymmetry can be different even between species with a similar G+C content. Indeed, in the *Phallusia *genus the GC-skew at P3 is quite different between the two species (0.13 and 0.36), although the G+C content is almost the same (see Table 4).

The high variability of base composition in ascidians has a strong influence on synonymous codon usage and even on amino acid composition of mitochondrial proteins. Thus, leucine is the most abundant amino acid in ascidians (average 15.1% ± 0.7%) as in other metazoan mt proteins but the usage of the two codon families (CTN and TTR) is very different between ascidian species (see standard deviation: CTN = 4.8% ± 3.4%; TTR = 10.2% ± 2.9%). Similarly, AGR and GGN glycine codon families are used differently between species (see standard deviation: GGN = 6.2% ± 4.8%; AGR = 4.7% ± 2.1%), as consequence of the different G content. Strong differences in amino acid abundance are also observed for amino acids encoded by quartet codons, such as Ala-GCN (3.9% ± 1.4%) and Val-GTN (9.7% ± 3.1%), and some amino acid encoded by AT-rich codons (Asn-AAY, Lys-AAR, and Ile-ATY). In all these cases, the standard deviation of the amino acid content exceeds 30% of the average value (data not shown).

### Protein-coding genes

As already reported, *cox2 *and *cob *genes overlap in both *Phallusia *species and in all other tunicate mtDNAs so far sequenced (length overlap from 11 to 29 bp). This gene overlap could be responsible of the linkage between *cox2 *and *cob *(Figure 1), preventing gene rearrangements involving these two genes. EST analyses have demonstrated that *cox2*-*cob *genes are transcribed to a single bicistronic mature mRNA in *C. intestinalis sp. A *and in *H. roretzi *[38]. Thus, a bicistronic mature mRNA for *cox2*-*cob *can be hypothesized even in *Phallusia*. The conservation of this overlap could be due to the need to produce a bicistronic mRNA or to a functional constraint at protein level, such as the need to preserve a specific amino acid pattern in the upstream ORF (i.e. in *cox2*). A careful analysis of the overlap region shows that the sequence included in both ORFs encodes for an amino acid region not conserved at the C-terminal region of COX2, but well conserved at the N-terminal of the COB protein (the downstream gene), where an arginine is present in all analysed deuterostomes. Thus, a functional constraint at protein level for this gene overlap can be excluded.

In *P. mammillata*, there are two additional cases of overlapping ORFs: *nad1*-*nad2*, with a 14 bp overlap, and *nad4L*-*cox3*. In the latter case, a complete TAA stop codon for *nad4L *is located 233 bp inside *cox3*, giving rise to a NAD4L protein showing a longer C-terminal region with no similarity to other tunicate NAD4L. Assuming the existence of an incomplete stop codon, a NAD4L with no frame overlap and length consistent to the homolog of *P. fumigata *can be inferred [see Additional file 1]. As in the case of *cox2*-*cob*, a frame overlap for *nad4L*-*cox3 *implies the presence of a bicistronic mature mRNA, whereas an incomplete stop codon implies the synthesis of distinct polyadenylated transcripts and the presence of a polyadenylation signal downstream *nad4L*. It is interesting that even in *H. roretzi*, *nad4L *gene exhibits a complete stop codon well inside the downstream tRNA gene, that is 76 bp downstream of the predicted incomplete stop codon [38].

Most *Phallusia *genes are inferred to have a complete termination codon, except for *cob *of *P. fumigata *and *nad4L *of *P. mammillata *[see Additional file 1]. Such abbreviated stop codons are assumed to be completed to a TAA codon during the process of maturation and polyadenylation of precursor polycistronic transcripts, as demonstrated by experimental data in mammals [46] and by "in silico" analyses of mt ESTs in two ascidians [38]. In these cases, a complete stop codon is often located a few bases downstream of the incomplete one, as a mechanism to prevent a translational readthrough in case of incorrect transcript maturation [47]. Curiously, this situation does not hold in *Phallusia*, given that complete stop codons are well inside the downstream gene in both *cob *and *nad4L *genes where an incomplete stop codon has been found (position 20 of the downstream *trnF *gene for *cob *of *P. fumigata*, and position 233 of the downstream *cox3 *gene for *nad4L *of *P. mammillata*).

As expected from the high G content (Table 4), the preferred stop codon is TAG in *P. fumigata *(10 genes) while in *P. mammillata *TAG and TAA are equally used as stop codons [see Additional file 1]. Similarly, the preferred start codon is GTG (8 in *P. fumigata *and 5 in *P. mammillata*), and most inferred start codons end with a G (12 in *P. fumigata*, and 9 in *P. mammillata*) [see Additional file 1].

### Cytochrome b

**Figure 4:**
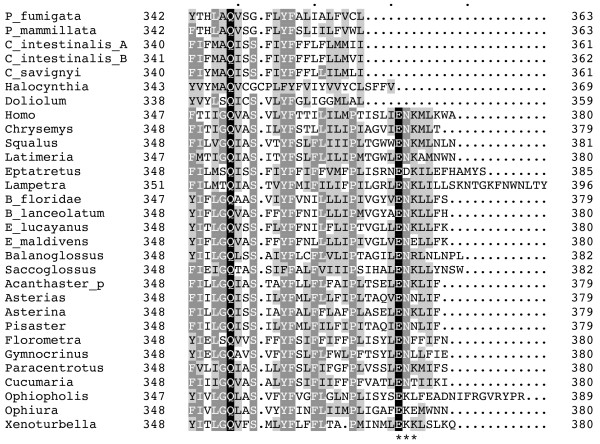
**Alignment of the C-terminal region of cytochrome b**. All available tunicate species and representatives of remaining deuterostomes are shown. Identical and conserved residues are shown by reverse contrast with black and dark gray background, respectively. Similar residues are indicated by light gray background. The "ENK" consensus sequence of non-ascidian proteins is highlighted by asterisks. Numbers refer to amino acid position. Species classification and AC number of the analysed sequences are listed in Additional file 2.

The cytochrome b protein (COB) of *Phallusia *and other tunicates is significantly shorter than in other deuterostomes, indeed the COB mean length is 363 ± 3 amino acids in tunicates, against 381 ± 4 in other deuterostomes (see Additional file 2 for species list). This length difference is due to the lack of a carboxy-terminal sequence of 10–24 amino acids, which is highly conserved from vertebrates to echinoderms and even in *Xenoturbella*. The lost region include the ENK consensus sequence, with a negatively charged amino acid (E) conserved in all analysed deuterostomes, and a positively charged amino acid in the last position of the consensus (K or R in 15 over 23 sequences) (Figure 4). COB is a membrane-spanning protein whose structure has been widely studied both at evolutionary and functional level [48-50]. This protein consists of three functional domains, each evolving at a different rate: an intermembrane, a transmembrane, and a matrix domain. The intermembrane domain is the one that evolves most slowly, because it contributes structurally to the Qo redox center. The transmembrane domain consists of eight hydrophobic helices, including those binding the two heme prosthetic groups [51], and is characterized by a preponderance of conservative amino acid changes [48]. The matrix domain includes three extramembrane loops protruding into the mitochondrial matrix, the N-terminal and C-terminal segments of the protein. This domain is poorly conserved, with amino acid replacements fitting neutral evolutionary expectations and reflecting the relative lack of function of this region [52]. Moreover, a previous analysis of more than 800 COB from bacteria to mammals has shown that the last 100 C-terminal residues of this protein are highly variable and that the immediately upstream H helix is one of the two least conserved transmembrane helices [50]. The COB fragment lost in tunicates is the C-terminal segment of the matrix domain, thus the tunicate protein terminates with the hydrophobic transmembrane H helix (Figure 4). The poor conservation of this region in COB of other organisms is compatible with a full functionality of this truncated protein. Moreover, the C-terminal segment following the H-helix is lost also in COB of trypanosomes [50], and greatly shortened or lost in some arthropods, nematodes and molluscs (data not shown).

### rRNA genes

*rrnS *is of similar size in the two *Phallusia *species (738 and 724 bp in *P. mammillata *and *P. fumigata*, respectively), whereas *rrnL *is significantly longer in *P. fumigata *than in *P. mammillata *because of the presence of an additional 120 bp-long sequence at the 5' end (1275 and 1147 bp in *P. mammillata *and *P. fumigata*, respectively). However, the additional *rrnL *sequence can be also considered as a non-coding region located upstream *rrnL *(i.e between *trnV *and *rrnL*). Indeed, a long NC region has been also annotated upstream *rrnL *in *C. intestinalis sp. A *and *sp. B*, where the 5'end of this gene was tentatively determined based on sequence conservation between orthologous genes of the three available *Ciona *species [33,34]. In general, the lack of transcripts and experimental data on mt rRNAs prevents the exact identification of the rRNA boundaries, and leaves open the possibility of the existence of non-coding regions flanking these genes.

The rRNA length of *Phallusia *species confirms a previous observation showing that tunicates have some of the shortest rRNAs among deuterostomes and even among metazoans [33], their size being comparable to the shortest rRNAs of nematodes and platyhelminthes [53]. Indeed, the average size of tunicate rRNAs is 695 ± 35 for *rrnS*, and 1134 ± 68 for *rrnL*, against an average rRNA size in other deuterostomes of 864 ± 33 for *rrnS*, and 1461 ± 93 for *rrnL *(species listed in Additional file 2).

### tRNA genes

The mtDNA of both *Phallusia *species lacks the tRNA-Asp gene (*trnD*). We sought this gene without success on both genome strands, even as an unusual cloverleaf structure with only a standard anticodon arm, or as a common cloverleaf structure with a non canonical Asp anticodon (5'-VYC-3' instead of 5'-GUC-3'). The absence of the *trnD *is inconsistent with the *Phallusia *codon usage, indeed the percentage of GAY codons for Asp is similar between *Phallusia *and other ascidians (1.83 ± 0.01% in *Phallusia *against 1.83 ± 0.13% in other ascidians). Moreover, the GAT codon is preferred over GAC, as expected from compositional data (see Table 4). The mitochondrial import of a nuclear-encoded tRNA-Asp could functionally compensate the lack of *trnD *in the mtDNA. Alternatively, it is possible that a highly modified mitochondrial *trnD*, with a transcript maturated through RNA-editing, has not been identified in the mtDNA because of its unusual secondary structure. Both mechanisms, tRNA import in mitochondria and tRNA-editing by anticodon modification, have been observed in marsupial mtDNA [54-56].

In both *Phallusia *species, three tRNAs (-Asn, -Ser(AGY), and -Cys) show an unusual cloverleaf structure [see Additional file 3 and Additional file 4].

In tRNA-Asn, the spacer between the DHU and anticodon (AC) stems is 2 nt long, instead of the canonical 1 nt [57]. This configuration also holds for tRNA-Asn of the stolidobranch *H. roretzi *and the thaliacean *D. nationalis *but not for other phlebobranches (i.e *Ciona*).

The tRNA-Ser(AGY) retains a DHU-arm with a stem 3 bp long and a large loop (11 nt long in *P. mammillata *and 15 nt *P. fumigata*), while in most metazoans the DHU-arm is replaced by a loop of variable size [57]. This peculiar cloverleaf structure has not been found in other tRNA-Ser(AGY) of analysed tunicates and deuterostomes [see Additional file 2], and it is probably a derived character of the *Phallusia *genus or of a narrow ascidian group.

The tRNA-Cys lacks the DHU arm, which is replaced by a loop 5 nt long, and shows several mismatches in stem base pairing compared to remaining tRNAs (4 total mismatches in *P. fumigata *and 7 in *P. mammillata*) [see Additional file 3 and Additional file 4]. Among analysed deuterostomes [see Additional file 2], this feature is shared by ascidians of the order Phlebobranchiata (*Ciona *and *Phallusia*) and by cephalochordates. Moreover, literature data report that the DHU arm of tRNA-Cys is sporadically lost also in vertebrates such as acrodont lizards [58] and the tuatara *Sphenodon punctatus *(Rhynchocephalia) [59]. Thus, current data suggest that the ancestral secondary structure of tRNA-Cys in deuterostome was the standard one, and that the DHU arm was lost independently in multiple lineages. Moreover, the non-canonical structure exhibited by tRNA-Cys even in some protostomes and flatworms [30,60-62] could indicate a tendency of this tRNA to better tolerate alterations of the cloverleaf structure.

It is remarkable that in ascidians unusual tRNA cloverleaf structures with lack/acquisition of the DHU-stem have been found in tRNA genes differing for the last nucleotide of the anticodon sequence (5'-GCU-3' in tRNA-Ser(AGY) and 5'-GCA-3' in tRNA-Cys). Exchanges in amino acid specificity of these tRNAs by a single nucleotide mutation in the anticodon or relaxed constraints related to the anticodon specificity could explain this observation.

As previously reported, the mtDNA of *P. fumigata *presents two *trnI*, and a *trnX *gene encoding for a tRNA with uncertain anticodon specificity (Figure 1).

The two copies of *trnI *are about 6 kb apart and differ only by two nucleotides at the 3' end of the amino acid (AA) stem: *trnI-1 *is located in a position similar to the orthologous gene of *P. mammillata *(upstream *rrnS*, see Figure 1), thus it is likely to be the ancestral *trnI *gene; *trnI-2 *is in the middle of a large duplicated sequence, embedded between two non-coding regions (see Figure 2), thus probably arose by a recent gene duplication event. The region including *trnI-2 *reported in Figure 2 could be the result of a "duplication-random gene loss" mechanism, with the gene loss step not having occurred yet.

**Figure 5:**
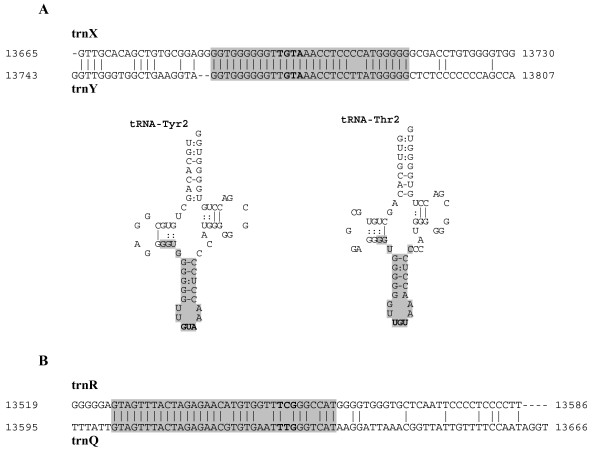
**tRNA genes of *P. fumigata *sharing a long and almost identical sequence (length ≥ 30 bp; identity ≥ 85%)**. (A) Similarity between *trnX *and *trnY*, and alternative secondary structures of the tRNA encoded by *trnX *(tRNA-Tyr2: position 13667–13730; tRNA-Thr2: position 13665–13729). (B) Similarity between *trnR *and *trnQ*. A gray background indicates almost identical sequences. Anticodon sequences are in bold. Canonical and G-U base pairing are differently indicated in the tRNA structure.

The *trnX *gene is part of the tRNA cluster *trnR*-*trnQ*-*trnX*-*trnY*, and contains a sequence 30 bp-long showing 93% identity to a portion of the downstream *trnY *gene (Figure 5). With slightly different boundaries, the *trnX *gene can be folded into two canonical cloverleaf secondary structures with a distinct amino acid specificity (Figure 5): a tRNA isoacceptor for Tyr (5'-GUA-3' anticodon), as expected by the sequence similarity to *trnY*; and a tRNA isoacceptor for Thr, with the 5'-UGU-3' anticodon overlapped to the anticodon of the alternative tRNA-Tyr structure. In both cloverleaf structures the anticodon is preceded by U and followed by the purine A, and there are only few mismatches in the stems (see Figure 5), thus both structures are compatible with functional tRNAs. Moreover, the low sequence similarity of *trnX *of *P. fumigata *with both *trnY *and *trnT *of *P. mammillata *suggests that *trnX *is not the true ortholog of these genes but a newly acquired gene (data not shown).

In *P. fumigata *a similarity like that observed between *trnX *and *trnY *is found also in the upstream gene pair *trnR*-*trnQ *(Figure 1). Indeed, *trnR *contains a sequence 34 bp-long showing 85% identity to the downstream *trnQ *gene, including a 17 bp-long identical sequence (Figure 5). In other ascidian mtDNAs, the genes *trnR *and *trnQ *do not share any identical long sequence (i.e. > 7 bp), however the *trnR*-*trnQ *gene arrangement is conserved in all available ascidians, except for *P. mammillata*. These data could suggest that an alloacceptor gene recruitment mechanism [63,64] gave origin to these tRNA genes in *P. fumigata*, consistent with the existence of a single nucleotide difference between the anticodons of *trnR *(UCG) and *trnQ *(UUG). However, evolutionary reconstructions based on tRNA sequence similarity are unable to support this hypothesis, because of the low resolution of most nodes of the tree (data not shown).

## Conclusion

The newly determined mtDNAs of *Phallusia *species and the comparative data reported here highlight the strong differences in mtDNA evolutionary dynamics between ascidians and remaining chordates.

Apart from the genetic code [10], our comparative analyses identify and/or confirm some mt features as distinctive of ascidians, and possibly of all tunicates, that is: same transcriptional polarities of all genes; presence of two *trnM *genes with different anticodon specificity; short rRNAs, comparable in size to the shortest mt rRNAs of nematodes and platyhelminthes; and a "truncated" cytochrome b protein lacking the C-terminal tail, which usually protrudes into the mitochondrial matrix.

The most striking ascidian features is the high frequency of gene order rearrangements, which is observed even in congeneric ascidians of two distinct genera (*Phallusia *and *Ciona*) and is not restricted to the tRNA genes, which often show an unstable mt genomic position. This extreme plasticity may represent a valuable source of data to study the mechanisms of gene rearrangement. Indeed, the analysis of gene order variability at low taxonomic distances increases the probability of identifying intermediary states of the rearrangement process and/or of observing ongoing rearrangement events. On the other hand, rapid gene rearrangements could impair the usage of gene order as phylogenetic character at high taxonomic level (because of a higher probability of gene order convergence in separate lineages) but will promote its usage to investigate speciation events [34].

Changes in tRNA secondary structure, tRNA content, length and position of NC regions, and compositional properties constitute an additional source of variability in ascidian mtDNA. In particular, the variability of NC regions even at intra-genus level suggests that regulatory elements controlling replication and transcription, commonly assumed to be located in NC regions, can be taxon-specific or extensively overlapped to coding sequences. Of course, the functional importance and reliability of the above-mentioned features as "ascidian mitochondrial signatures" need to be further investigated in a wider ascidian sample.

## Methods

The specimens of *Phallusia mammillata *and *Phallusia fumigata *were collected on the coast of Liguria, Tirrenian Sea. Total DNA was extracted from siphon muscles of a single individual using the Puregene Tissue kit (Gentra Systems) following the manufacturer's protocol. For both species, the complete mtDNA was amplified by long-PCR in several overlapping fragments, in order to avoid the amplification of short mitochondrial pseudogenes sometimes present in the nuclear genome (Numts). Long-PCR was carried out with Expand High Fidelity PCR System (Roche Applied Science), Expand Long Template PCR System (Roche Applied Science), or LA-Taq enzyme (TaKaRa), using several heterologous primers designed on the most conserved mt ascidian sequences: large subunit rRNA (*rrnL*), cytochrome b (*cob*) and cytochrome c oxidase subunit (*cox1*, *cox2*, and *cox3*) genes. Given the absence of information on the mtDNA gene order in *Phallusia *species, several combinations of these heterologous primers were initially used in PCR reactions, and only those reactions that gave a bright single band during electrophoretic analysis were further processed. Species-specific primers were designed on the sequences of the first successfully amplified and sequenced fragments, and used to amplify the remaining regions of the genome. In summary, the mtDNA of *P. mammillata *was amplified in three long overlapping fragments (size range: 2.5 – 9.4 kb), whereas the mtDNA of *P. fumigata *was amplified in five overlapped fragments (size range: 1.2 – 5 kb). Moreover, in both species an additional fragment was amplified to confirm the overlap surrounding the *rrnL *gene. Amplified fragments, primers sequences and PCR conditions are reported in Additional file 5. Fragments shorter than 3 kb were directly sequenced, after purification with the Montage PCR Filter Units (Millipore), using a primer walking strategy (MWG Sequencing Service). Longer fragments were re-amplified in short overlapping segments (information available on request), and then sequenced by primer walking.

The complete mtDNA sequences of *P. mammillata *and *P. fumigata *were deposited at EMBL database under accession numbers AM292320 and AM292602, respectively.

Protein-coding gene annotations were based on sequence similarity to orthologous genes of ascidians. ATG and non-standard initiation codons reported in Wolstenholme [6] were considered as reliable start codons. The start of a protein-coding gene was inferred as the initiation codon that does not cause overlap with the upstream gene and maximizes the similarity between proteins of the two *Phallusia *species. Whenever possible, incomplete stop codons were inferred in order to avoid overlap with the downstream genes.

Transfer RNA genes were identified by their potential cloverleaf secondary structures. The tRNAscan-SE v1.23 program (UNIX version) [65] was locally run with relaxed parameters specifically defined for ascidians (used options: -O -y -X 0 -L 10 -g ascidiancode); the PatSearch program [66] was used to search for very unusual tRNA structures, such as those lacking an arm. All putative tRNAs were manually checked, considering the reliability of secondary structure and the alignment with known ascidian tRNAs. Thus, transfer RNA identity was specified by anticodon sequence, and sequence similarity.

Boundaries of rRNA genes of small and large ribosomal subunit (*rrnS *and *rrnL*, respectively) were inferred from the boundaries of flanking genes.

Compositional features and codon usage similarities were calculated with Codontree [67]. The GC- and AT-skews, which indicate compositional differences between the two strands, were calculated according to the formulae of Perna and Kocher [68]

Exact direct repeats longer than 10 bp were identified using the Reputer program package [69].

Comparative analyses were carried out on complete mtDNAs of deuterostomes [see Additional file 2]. In addition to *Phallusia*, 49 sequences were analysed, including 10 representatives of Vertebrata, and all available species of Agnatha (4 species), Cephalochordata (11), Tunicata (5), Hemichordata (2), Echinodermata (16), and Xenoturbellidae (1).

Alignments of the 13 mitochondrial proteins were performed with CLUSTAL W v1.82 [70] and manually revised, then the equivalent nucleotide (nt) alignments were "back-aligned" from the protein data. Transfer RNA genes were aligned based on their secondary structure.

Gene order rearrangements were analysed on two datasets, with or without tRNA genes, and the extra genes of *P. fumigata *(*trnI-2 *and *trnX*) were excluded from all analyses. The GeneSyn program [71] allowed the detection of gene strings conserved in chordate mtDNAs. The BPAnalysis program [72] was used to calculate the breakpoint distances between pairs of genomes with identical gene content (i.e. the number of gene adjacencies present in one genome but absent in the other). This breakpoint measure was selected because it makes no assumptions about the rearrangement mechanisms. Given the differences in gene content between species and between datasets, a relative breakpoint distance was calculated dividing the breakpoint distance by the number of genes shared by each pair of analysed genomes.

The number of nonsynomymous (dN) and synonymous (dS) substitutions per site were calculated according to the codon substitution model of Goldman and Yang [73], using the Codeml program of the PAML v3.15 package [74]. The program was run on the gap-free concatenated alignment of the 13 protein-coding genes, with codon frequencies calculated from the average nucleotide frequencies at the three codon positions (option CodonFreq = 2), and maximum likelihood (ML) estimations in pairwise comparisons (option RunMode = -2). Sequence divergence of rRNA genes was calculated on gap-free alignments with the Baseml program (PAML v3.15 package) [74] according to the GTR nucleotide substitution model [75].

## Authors' contributions

FI amplified, sequenced and annotated the complete mtDNA of *P. mammillata*; FG amplified, sequenced and annotated the complete mtDNA of *P. fumigata*. In addition, FI and FG participated in the bioinformatics analyses. GP participated in the study design, and critically revised the manuscript. CG conceived the study, supervised the research, carried out most bioinformatics analyses and drafted the manuscript. All authors have provided critical reviews of the manuscript content, read and approved the final manuscript.

## Supplementary Material

Additional file 1 Length, start and stop codons of the mitochondrial protein-coding genes of the two *Phallusia *species. Length, start and stop codons of the mitochondrial protein-coding genes of the two *Phallusia *species. Click here for file

Additional file 2 Deuterostome mitochondrial genomes analysed in this study. Accession numbers of the deuterostome mtDNA sequences analysed in this study. Organism classification is also reported. Click here for file

Additional file 3 Putative secondary structures of tRNAs encoded by *Phallusia fumigata *mtDNA. Putative secondary structures of tRNAs encoded by *P. fumigata *mtDNA. Canonical and G-U base pairing are differently indicated. Yellow background indicates overlapped sequences belonging to adjacent tRNA genes. Nucleotides in square brackets indicate differences in tRNA-Ile2 compared to the reported tRNA-Ile1 sequence. Click here for file

Additional file 4 Putative secondary structures of tRNAs encoded by *Phallusia mammillata *mtDNA. Putative secondary structures of tRNAs encoded by *P. mammillata *mtDNA. Canonical and G-U base pairing are differently indicated. Yellow background indicates overlapped sequences belonging to adjacent tRNA genes. Click here for file

Additional file 5 Primer sequences and PCR conditions used to amplify the two *Phallusia *mtDNAs. Primer sequences and PCR conditions used to amplify the whole mtDNA of the two *Phallusia *species in several overlapped fragments. Click here for file
