# Supplementary material for: The mitochondrial genome of Phallusia mammillata and Phallusia fumigata (Tunicata, Ascidiacea): high genome plasticity at intra-genus level
Source: BMC Evol Biol. 2007 Aug 31;7:155. doi: 10.1186/1471-2148-7-155 (PMC2220002; doi:10.1186/1471-2148-7-155)
Supplement: Additional file 1 — Length, start and stop codons of the mitochondrial protein-coding genes of the two Phallusia species. Length, start and stop codons of the mitochondrial protein-coding genes of the two Phallusia species. [file 1471-2148-7-155-S1.doc]

**Table S2. Length, start and stop codons of the mitochondrial protein-coding genes of the two *Phallusia* species.**

|  | ***Phallusia mammillata*** | | | |  | ***Phallusia fumigata*** | | | |
| --- | --- | --- | --- | --- | --- | --- | --- | --- | --- |
|  | **Start** | **Stop** | **L (bp)** | **Spacer (bp)** ^a^ |  | **Start** | **Stop** | **L (bp)** | **Spacer (bp)** ^a^ |
| *atp6* | ATG | TAG | 639 | 15 |  | GTG | TAG | 639 | 6 |
| *atp8* | ATT | TAA | 96 | 1 |  | ATT | TAG | 96 | 28 |
| *cob* | ATG | TAG | 1092 | 2 |  | ATG | T | 1090 | 0 |
| *cox1* | GTG | TAG | 1530 | 0 |  | GTG | TAG | 1545 | 134 |
| *cox2* | GTG | TAA | 687 | -14 |  | GTG | TAA | 687 | -14 |
| *cox3* | GTG | TAA | 789 | 10 |  | TTG | TAG | 792 | 9 |
| *nad1* | ATA | TAG | 909 | -14 |  | GTG | TAG | 918 | 22 |
| *nad2* | ATA | TAA | 1014 | 3 |  | ATG | TAA | 1023 | 7 |
| *nad3* | ATG | TAA | 375 | 5 |  | GTG | TAG | 381 | 123 |
| *nad4* | GTG | TAG | 1392 | 0 |  | GTG | TAG | 1380 | 11 |
| *nad4L* | ATA | T | 256 | 3 |  | GTG | TAG | 270 | 9 |
| *nad5* | GTG | TAA | 1644 | 58 |  | ATG | TAG | 1653 | 1 |
| *nad6* | TTG | TAG | 474 | 7 |  | GTG | TAG | 480 | 104 |

^a^  Length of the non-coding region between the reported gene and the downstream gene. Negative values indicate gene overlap.
