# Supplementary material for: The mitochondrial genome of Phallusia mammillata and Phallusia fumigata (Tunicata, Ascidiacea): high genome plasticity at intra-genus level
Source: BMC Evol Biol. 2007 Aug 31;7:155. doi: 10.1186/1471-2148-7-155 (PMC2220002; doi:10.1186/1471-2148-7-155)
Supplement: Additional file 2 — Deuterostome mitochondrial genomes analysed in this study. Accession numbers of the deuterostome mtDNA sequences analysed in this study. Organism classification is also reported. [file 1471-2148-7-155-S2.doc]

**Table S1. Deuterostome mitochondrial genomes analysed in this study. Organism classification is also reported.**

| **Classification** | **Species** | **AC number** |
| --- | --- | --- |
| Vertebrata, Mammalia | *Homo sapiens* | AF347015 |
| Vertebrata, Mammalia | *Oryctolagus cuniculus* | AJ001588 |
| Vertebrata, Aves | *Gallus gallus* | X52392 |
| Vertebrata, Archosauria | *Alligator mississippiensis* | Y13113 |
| Vertebrata, Testudines | *Chrysemys picta* | AF069423 |
| Vertebrata, Squamata | *Dinodon semicarinatus* | AB008539 |
| Vertebrata, Amphibia | *Rana nigromaculata* | AB043889 |
| Vertebrata, Elasmobranchia | *Squalus acanthia* | Y18134 |
| Vertebrata, Neopterygii | *Gadus morhua* | X99772 |
| Vertebrata, Neopterygii | *Latimeria chalumnae* | U82228 |
| Agnatha, Hyperoartia | *Petromyzon marinus* | U11880 |
| Agnatha, Hyperoartia | *Lampetra fluviatilis* | Y18683 |
| Agnatha, Hyperotreti | *Myxine glutinosa* | AJ404477 |
| Agnatha, Hyperotreti | *Eptatretus burgeri* | AJ278504 |
| Cephalochordata | *Branchiostoma floridae* ^a^ | Y16474 |
| Cephalochordata | *Branchiostoma lanceolatum* | AB194383 |
| Cephalochordata | *Branchiostoma floridae* | AF098298 |
| Cephalochordata | *Epigonichthys lucayanus* | AB110092 |
| Cephalochordata | Epigonichthys maldivensis | AB110093 |
| Cephalochordata | Branchiostoma belcheri ^b^ | AB078191 |
| Cephalochordata | *Branchiostoma belcheri* (Genkai-2) | AB083383 |
| Cephalochordata | *Branchiostoma belcheri* (Akashi-1) | AB083384 |
| Cephalochordata | *Branchiostoma belcheri* (Akashi-1) | AB083385 |
| Cephalochordata | *Branchiostoma belcheri* (strain O4.6*)* ^c^ | AY932825 |
| Cephalochordata | *Branchiostoma japonicum* | DQ407722 |
| Tunicata, Ascidiacea, Stolidobranchiata | *Halocynthia roretzi* | AB024528 |
| Tunicata, Ascidiacea, Phlebobranchiata | *Ciona savignyi* | AB079784 |
| Tunicata, Ascidiacea, Phlebobranchiata | *Ciona intestinalis sp A* | AJ517314 |
| Tunicata, Ascidiacea, Phlebobranchiata | *Ciona intestinalis sp B* | AM292218 |
| Tunicata, Ascidiacea, Phlebobranchiata | *Phallusia fumigata* | This study |
| Tunicata, Ascidiacea, Phlebobranchiata | *Phallusia mammillata* | This study |
| Tunicata, Thaliacea | *Doliolum nationalis* | AB176541 |
| Hemichordata, Enteropneusta | *Balanoglossus carnosus* | AF051097 |
| Hemichordata, Enteropneusta | *Saccoglossus kowalevskii* | AY336131 |
| Echinodermata, Asteroidea | *Asterina pectinifera* | D16387 |
| Echinodermata, Asteroidea | *Pisaster ochraceus* | X55514 |
| Echinodermata, Asteroidea | *Luidia quinalia* | AB183558 |
| Echinodermata, Asteroidea | *Asterias amurensis* | AB183559 |
| Echinodermata, Asteroidea | *Astropecten polyacanthus* | AB183560 |
| Echinodermata, Asteroidea | *Acanthaster planci* | AB231475 |
| Echinodermata, Asteroidea | *Acanthaster brevispinus* | AB231476 |
| Echinodermata, Crinoidea | *Florometra serratissima* | AF049132 |
| Echinodermata, Crinoidea | *Gymnocrinus richeri* | DQ068951 |
| Echinodermata, Crinoidea | *Phanogenia gracilis* | DQ068952 |
| Echinodermata, Echinoidea | *Paracentrotus lividus* | J04815 |
| Echinodermata, Echinoidea | *Arbacia lixula* | X80396 |
| Echinodermata, Echinoidea | *Strongylocentrotus purpuratus* | X12631 |
| Echinodermata, Holoturoidea | *Cucumaria miniata* | AY182376 |
| Echinodermata, Ophiuroidea | *Ophiopholis aculeata* | AF314589 |
| Echinodermata, Ophiuroidea | *Ophiura lutkeni* | AY184223 |
| Xenoturbellidae | *Xenoturbella bocki* | DQ832701 |

^a^ This sequence, published as belonging to *B. lanceolatum*, actually belongs to *B. floridae* [1].

^b^ Sequence chosen as representative of *B. belcheri*.

^c^ The sequence of strain O4.6 was not considered given its unpublished status, and the high divergence from other individuals of *B. belcheri*.

**References**

1. Nohara M, Nishida M, Nishikawa T**: New complete mitochondrial DNA sequence of the lancele*t Branchiostoma lanceolat*um (Cephalochordata) and the identity of this species' sequenc**es*. Zoolog Sc*i 2005**,** 22(6):671-674.
