# Supplementary material for: The mitochondrial genome of Phallusia mammillata and Phallusia fumigata (Tunicata, Ascidiacea): high genome plasticity at intra-genus level
Source: BMC Evol Biol. 2007 Aug 31;7:155. doi: 10.1186/1471-2148-7-155 (PMC2220002; doi:10.1186/1471-2148-7-155)
Supplement: Additional file 3 — Putative secondary structures of tRNAs encoded by Phallusia fumigata mtDNA. Putative secondary structures of tRNAs encoded by P. fumigata mtDNA. Canonical and G-U base pairing are differently indicated. Yellow background indicates overlapped sequences belonging to adjacent tRNA genes. Nucleotides in square brackets indicate differences in tRNA-Ile2 compared to the reported tRNA-Ile1 sequence. [file 1471-2148-7-155-S3.pdf]

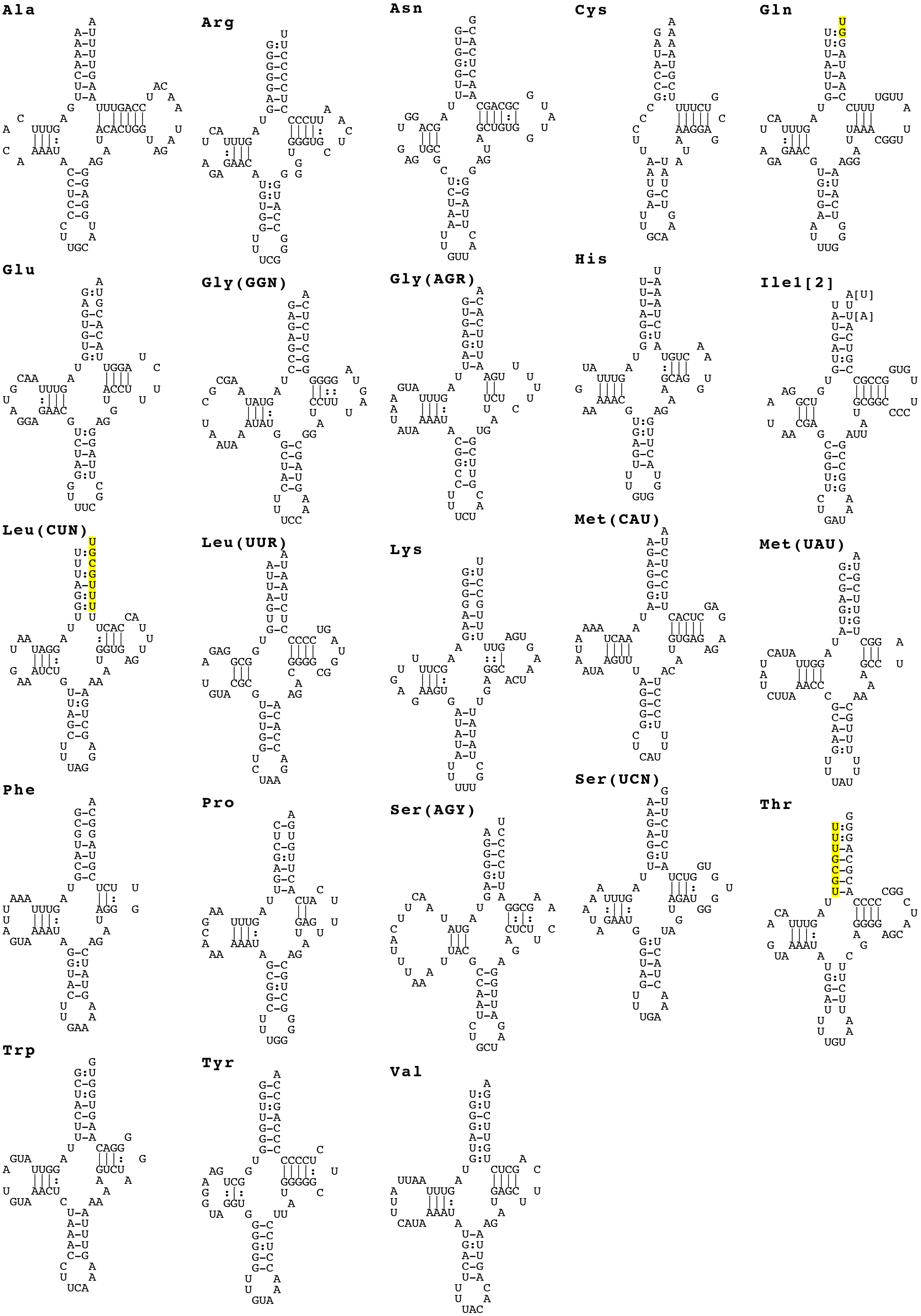
Ala
Arg
Asn
Cys
Gln
Glu
Gly(GGN)
Gly(AGR)
His
Ile1[2]
Leu(CUN)
Leu(UUR)
Lys
Met(CAU)
Met(UAU)
Phe
Pro
Ser(AGY)
Ser(UCN)
Thr
Trp
Tyr
Val
