# Supplementary material for: The mitochondrial genome of Phallusia mammillata and Phallusia fumigata (Tunicata, Ascidiacea): high genome plasticity at intra-genus level
Source: BMC Evol Biol. 2007 Aug 31;7:155. doi: 10.1186/1471-2148-7-155 (PMC2220002; doi:10.1186/1471-2148-7-155)
Supplement: Additional file 5 — Primer sequences and PCR conditions used to amplify the two Phallusia mtDNAs. Primer sequences and PCR conditions used to amplify the whole mtDNA of the two Phallusia species in several overlapped fragments. [file 1471-2148-7-155-S5.doc]

**Table S3: Primer sequences and PCR conditions used to amplify the whole mtDNA of the two *Phallusia* species in several overlapped fragments.**

| P. mammillata | |  |  |  |  |  |  |  |
| --- | --- | --- | --- | --- | --- | --- | --- | --- |
| Primer ^a^ | Sequence | **Tm ^b^** | **Amplified fragment ^c^** | **Name** | **Size**  **(kb)** | **Annealing temp.** | **Extension (time/temp.)** | **Enzyme ^d^** |
| ux2F | GYAGTTRGDCAYCARTGATATTG | 57.7 | cox2-rrnL | ux2F-u16R | 2.5 | 50°C | 1'30'' / 72°C | E-HiFi |
| u16R | TAARYCGACATCGAGGTCRTAA | 57.5 |  |  |  |  |  |  |
| 16F | AACCYYAGGGATAACAGCGC | 59.4 | rrnL-cox3 | 16F-ux3R | 3.1 | 54°C | 2'5'' / 68°C | E-HiFi |
| ux3R | TCWCGWWCAACATCMCGYCAYCA | 61.5 |  |  |  |  |  |  |
| pmx3F | GGACGGATCTCCTTGGCCCTTAATCGG | 69.5 | cox3-cox2 | pmx3F-pmx2R | 9.4 | 62°C | 20' / 68°C | LA-T |
| pmx2R | CGTCCGGGGACGCAATCAACCT | 65.8 |  |  |  |  |  |  |
| pm16F | GGAGACTCTGTTGTTGGGGAA | 59.8 | rrnL-nd2 ^e^ | pm16F-pmn2R | 2 | 54°C | 2' / 72°C | E-HiFi |
| pmn2R | ATTCCCCCCACCACCACAGAC | 63.7 |  |  |  |  |  |  |

| P. fumigata | |  |  |  |  |  |  |  |
| --- | --- | --- | --- | --- | --- | --- | --- | --- |
| **Primer** | Sequence | **Tm ^b^** | **Amplified fragment ^c^** | **Name** | **Size**  **(kb)** | **Annealing temp.** | **Extension (time/temp.)** | **Enzyme ^d^** |
| px2F | CAYCARTGGTACTGRAGYTAYGA | 59.8 | cox2-cob | px2F-pcyR | 1.2 | 54°C | 2'30'' / 72°C | E-HiFi |
| pcyR | AAAGTACCACTCAGGYTTRATRTG | 58.4 |  |  |  |  |  |  |
| pmcbF | GGCCTTATTTCGGGGTTAAGG | 59.8 | cob-cox3 | pmcbF-lg2R | 5 | 50°C | 4' / 68°C | E-Long |
| lg2R | CAATACCAAATCGCACACTC | 55.3 |  |  |  |  |  |  |
| pmx3F | GGACGGATCTCCTTGGCCCTTAATCGG | 69.5 | cox3-cox1 | pmx3F-ux1R | 2 | 48°C | 3' / 68°C | E-HiFi |
| ux1R | ATAAGCTCGWGAATCHACATC | 54.6 |  |  |  |  |  |  |
| ux1F | CCDGATATRGCKTTYCCTCG | 59.4 | cox1-rrnL | ux1F-u16R | 4.8 | 52°C | 10' / 68°C | E-HiFi |
| u16R | TAARYCGACATCGAGGTCRTAA | 57.5 |  |  |  |  |  |  |
| 16F | AACCYYAGGGATAACAGCGC | 59.4 | rrnL-cox2 | 16F-gx2R | 4.4 | 52°C | 3' / 72°C | E-HiFi |
| gx2R | AAYTCAACATGGATGGGYATRAA | 56.2 |  |  |  |  |  |  |
| f16F | GACRAAAAGACCCTAGGTGGTT | 59.3 | rrnL-nad5 ^e^ | f16F-pn5R | 3.1 | 52°C | 2'30" / 72°C | E-Long |
| pn5R | GCTGTGGATAAACACCCCA | 56.7 |  |  |  |  |  |  |

^a^ Primer beginning with “p” or “f” are species-specific primers designed on early *Phallusia* mt sequences obtained using heterologous primers (remaining primers of the table).

^b^ Tm: melting temperature of primers, in **°C**

^c^ Fragment name is based on genes located at the ends of the fragment itself

^d^ E-HiFi: Expand High Fidelity PCR System (Roche Applied Science); E-Long: Expand Long Template PCR System (Roche Applied Science); LA-T: LA-Taq enzyme (TaKaRa).

^e^ Fragments amplified to confirm a sequence overlap.
